# Real‐World Experience With Plasma PTAU217 as a Screening Tool of Patients at Risk of Alzheimer's Disease in a Memory Clinic

**DOI:** 10.1002/alz70856_101897

**Published:** 2025-12-24

**Authors:** Maria Capdevila, Raquel Puerta, Laura Montrreal, Álvaro Muñoz‐Morales, Clàudia Olivé, Itziar de Rojas, Pablo García‐González, Marta Martinez, Adelina Orellana, Natalia Tatinya, Montserrat Alegret, Pilar Sanz, Victoria Fernández, Sergi Valero, Marta Marquié, Mercè Boada, Agustin Ruiz, Amanda Cano

**Affiliations:** ^1^ Ace Alzheimer Center Barcelona, Barcelona, Spain; ^2^ Ace Alzheimer Center Barcelona‐Universitat Internacional de Catalunya, Barcelona, Spain; ^3^ University of Texas Health Science Center, San Antonio, TX, USA; ^4^ Ace Alzheimer Center Barcelona, Barcelona, Barcelona, Spain

## Abstract

**Background:**

With the approval of Disease Modifying Drugs for Alzheimer's disease (AD), emerging clinical trials and unknowns in etiology, the clinical use of plasma biomarkers is increasingly becoming a reality. Here we explore the implementation of plasma pTau217 as a screening tool for patients at risk of AD in the real‐world practice of an experienced memory clinic.

**Methods:**

The study cohort encompassed 2353 paired cerebrospinal fluid (CSF) and plasma samples collected during 8 years at Ace Alzheimer Center Barcelona memory clinic from real‐world patients with subjective cognitive decline (SCD), mild cognitive impairment (MCI), AD dementia and other dementias. Plasma pTau181 and pTau217 were measured using the Lumipulse G1200 automatic platform (Fujirebio Inc.). The study cohort was divided in testing and validation cohorts for the analysis.

**Results:**

Plasma pTau217 significantly correlated (*p* <0.0001) with its CSF homonymous in all phenotypes except the other dementias. ROC curve only defined by A+T+ *vs* A‐T‐ showed an AUC=0.97, *p* <0.0001, sensitivity 90.58% and specificity 94.46%. Plasma pTau217 showed a clear improvement against plasma pTau181 in the preclinical stage, not instead in the most advanced AD continuum phase. Moreover, plasma pTau181 levels were more affected by eGFR than plasma pTau217. MRI analysis showed that plasma pTau217 positively correlated with the white matter hypointensities and lateral ventricles volumes (rho>0.40, *p* <0.0001) and negatively correlated with the amygdala and hippocampus volumes (rho<‐0.41, *p* <0.0001). Survival analysis showed that MCI with the plasma pTau217 value above the pre‐defined cut off (0.19 pg/ml) exhibited a 4.75 higher risk to convert to AD dementia than patients under the cut off value. Validation cohort confirmed all these findings.

**Conclusion:**

Plasma pTau217 exhibits better results than plasma pTau181 in the preclinical stages of AD continuum. All results together suggest that plasma pTau217 is robust enough to be implemented as a screening tool of AD risk in the memory clinics. However, since plasma biomarkers are not still approved by the international guidelines for their use in the clinical practice, a CSF/PET confirmatory test is mandatory required. Further studies are needed to obtain more clinical data and improve the biomarker's performance to finally substitute CSF/PET biomarkers as diagnostic tests

